# Atypical Injuries of the Elbow in Children: A Case Series

**DOI:** 10.7759/cureus.84504

**Published:** 2025-05-20

**Authors:** Supratim Roy, Aliasgar Moaiyadi, Bhavesh Patidar, Akshay V Rode, Prithviraj Deshmukh

**Affiliations:** 1 Orthopaedics, Mahatma Gandhi Institute of Medical Sciences (MGIMS), Wardha, IND

**Keywords:** capitellum, monteggia, neglected elbow, pediatrics, rare elbow injuries

## Abstract

Fractures occurring in the elbow region of children and adolescents pose a notable challenge for orthopedic surgeons due to the complex anatomy and developmental stages of the humerus. Capitulum fractures, a rare form of elbow injury, are classified into three types according to the Bryan and Morrey classification system. Monteggia fractures, a rare occurrence in children, involve a combination of ulna break and radial head dislocation, with Monteggia equivalent fractures expanding upon traditional classifications. The distinction lies in Monteggia equivalent fractures (MEFs) not always involving proximal radioulnar joint separation. Pediatric cases are further complicated by immature radiocapitellar epiphysis, potentially leading to misdiagnosis or neglect due to concealed joint presentation. This article highlights these rare elbow injuries, emphasizing the need for specialized attention and management.

This article presents a case series of four patients with rare elbow injuries that were diagnosed and treated operatively and followed until union with acceptable functional outcome, highlighting the need for early diagnosis and treatment in such cases.

Fractures around the elbow in children present diagnostic and therapeutic challenges due to complex anatomy. Capitulum and Monteggia equivalent fractures require careful management to prevent complications. Prompt recognition and treatment, be it closed reduction or open reduction, are crucial for optimal outcomes. Further research on neglected lateral humeral condyle (LHC) fractures is needed to inform clinical decisions.

## Introduction

Elbow fractures in the pediatric population pose complex diagnostic and therapeutic challenges for orthopedic specialists [1]. The complexity of the humerus' distal anatomy is compounded by the presence of multiple ossification centers that appear at various stages of a child's bone development. This complexity is a key factor in the difficulty of treating injuries in this region [2]. Fractures of the capitulum, although uncommon, making up less than 1% of all elbow-related injuries, have been systematically categorized by Bryan and Morrey into three distinct types. Type 1 includes coronal shear fractures, type 2 encompasses a small layer of bone beneath the cartilage, and type 3 is often found alongside fractures of the radial head [3]. Monteggia fractures, characterized by a fracture in the proximal third of the ulna and a dislocation of the radial head, are considered rare among children, representing only 0.4% of forearm fractures within this demographic [4]. Recent years have seen an increase in the types of injuries classified as Monteggia equivalent fractures, thereby extending the scope of the Bado classification [5]. A notable distinction is that Monteggia equivalent fractures do not typically result in dissociation at the proximal radioulnar joint [6]. In children, the diagnosis is further complicated by an underdeveloped radiocapitellar epiphysis, and the pliable joint increases the propensity for the joint to subluxate easily. Such instances of this nature can lead to misdiagnoses or an oversight, especially when abnormalities in the radiocapitellar joint or ulnar bowing are not evident in X-ray images [6].

Therefore, this article aims to highlight the unique considerations and management strategies required for these infrequent but significant elbow injuries in children.

## Case presentation

Case 1

**Figure 1:**
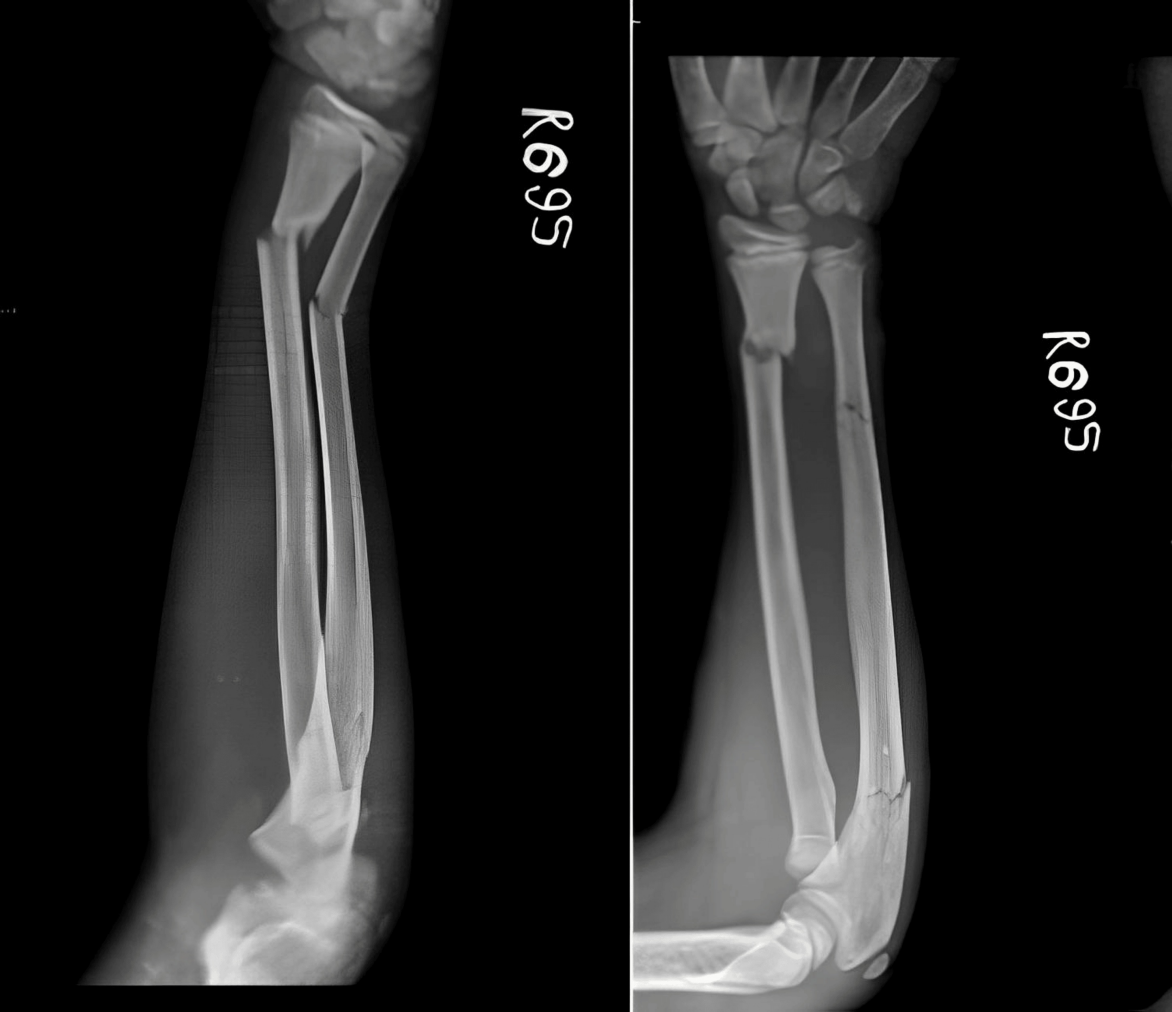
Preoperative X-ray of case 1 showing distal fracture of the radius with segmental ulna fracture and radial head fracture

A 13-year-old boy came to the casualty with his right forearm in pain and swollen, having fallen from a height during play about five hours earlier. Despite the apparent deformities of the wrist and elbow joints and restricted movement, the radioulnar artery pulse was palpable, and the boy could dorsiflex his fingers. Initial X-rays showed distal fractures of both the radius and ulna and a proximal fracture of the ulna, as well as a displaced radial head fracture (Figure 1).

**Figure 2:**
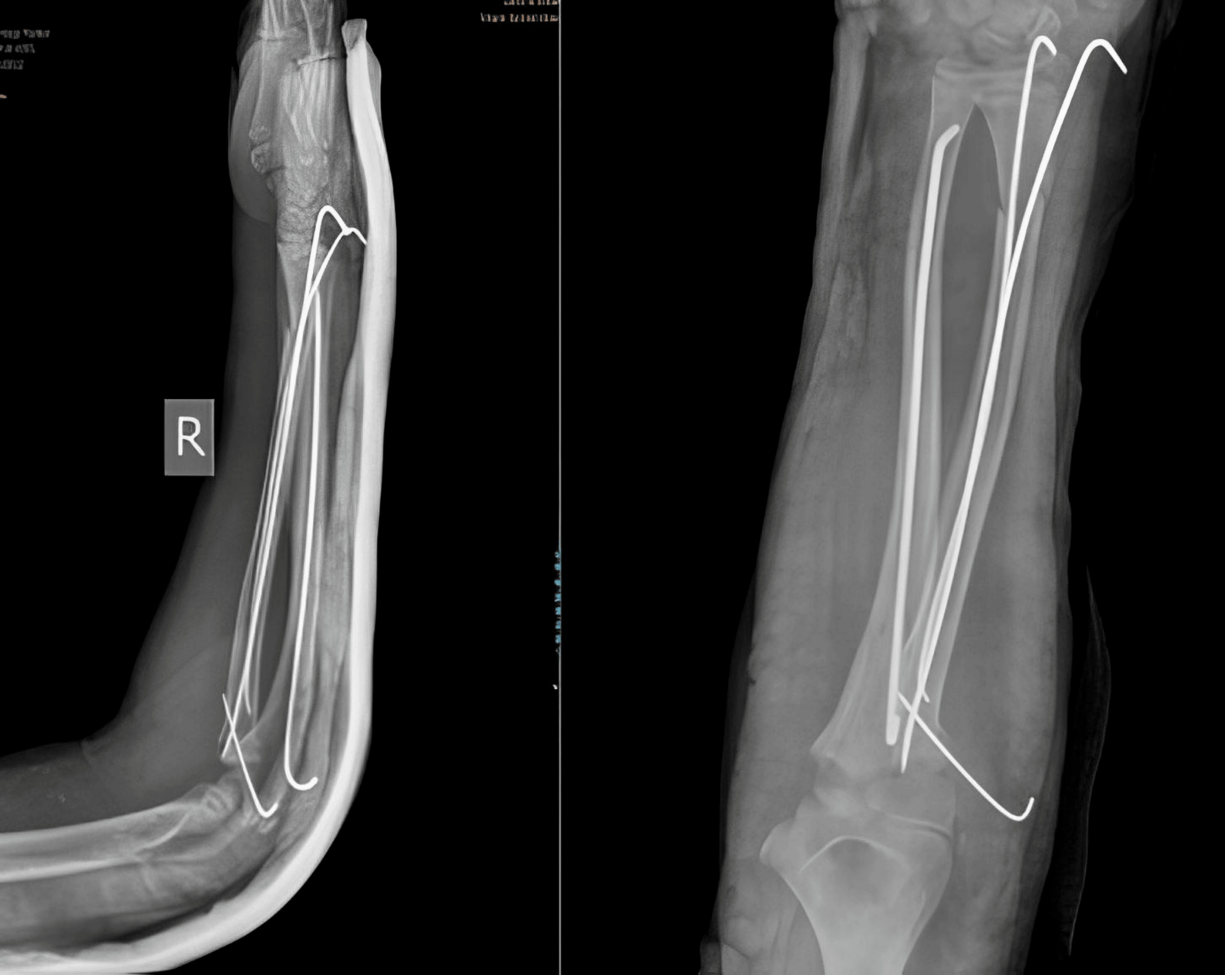
Postoperative X-ray of case 1

**Figure 3:**
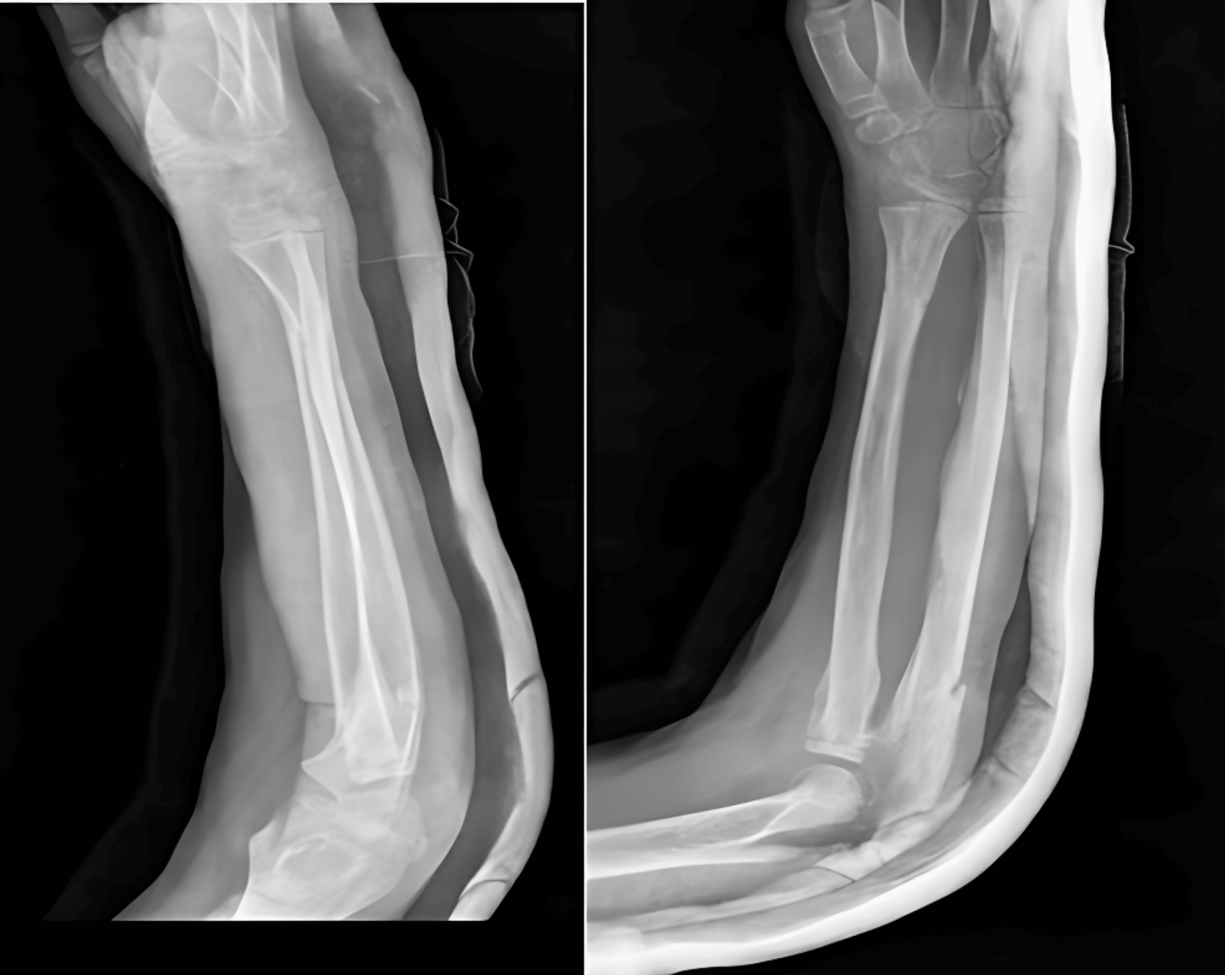
Postoperative X-ray of case 1 after implant removal

Initial attempts at manual reduction were followed by immobilization with an above-elbow slab, but follow-up imaging indicated the need for further intervention due to inadequate reduction of the fracture. About two days post-injury, the boy underwent a surgical procedure under brachial plexus block. The surgery involved closed reduction and fixation of the fractures using long, flexible Kirschner wires (K-wires) placed carefully to avoid growth plate damage and guided by intraoperative fluoroscopy. To address the displaced radial head fracture, the forearm was subsequently supinated, and the elbow joint was flexed to facilitate reduction. Additional K-wires were inserted, passing through the head of the radius and engaging with the shaft, effectively correcting the displaced fracture of the radial head (Figure 2). After the surgery, the forearm was supported again with a splint, and the patient was allowed to go home after three days in the hospital. Follow-ups continued on an outpatient basis, and after two months, during which the boy engaged in rehabilitative exercises, the supportive hardware was removed (Figure 3). The patient's dedication to physical therapy paid off; he regained considerable functionality, with an 80° range of pronation, 70° supination, and a 100° range of motion in the elbow at six months. His functional outcome, as quantified by a Broberg and Morrey score, was a satisfactory 88 points.

Case 2

**Figure 4:**
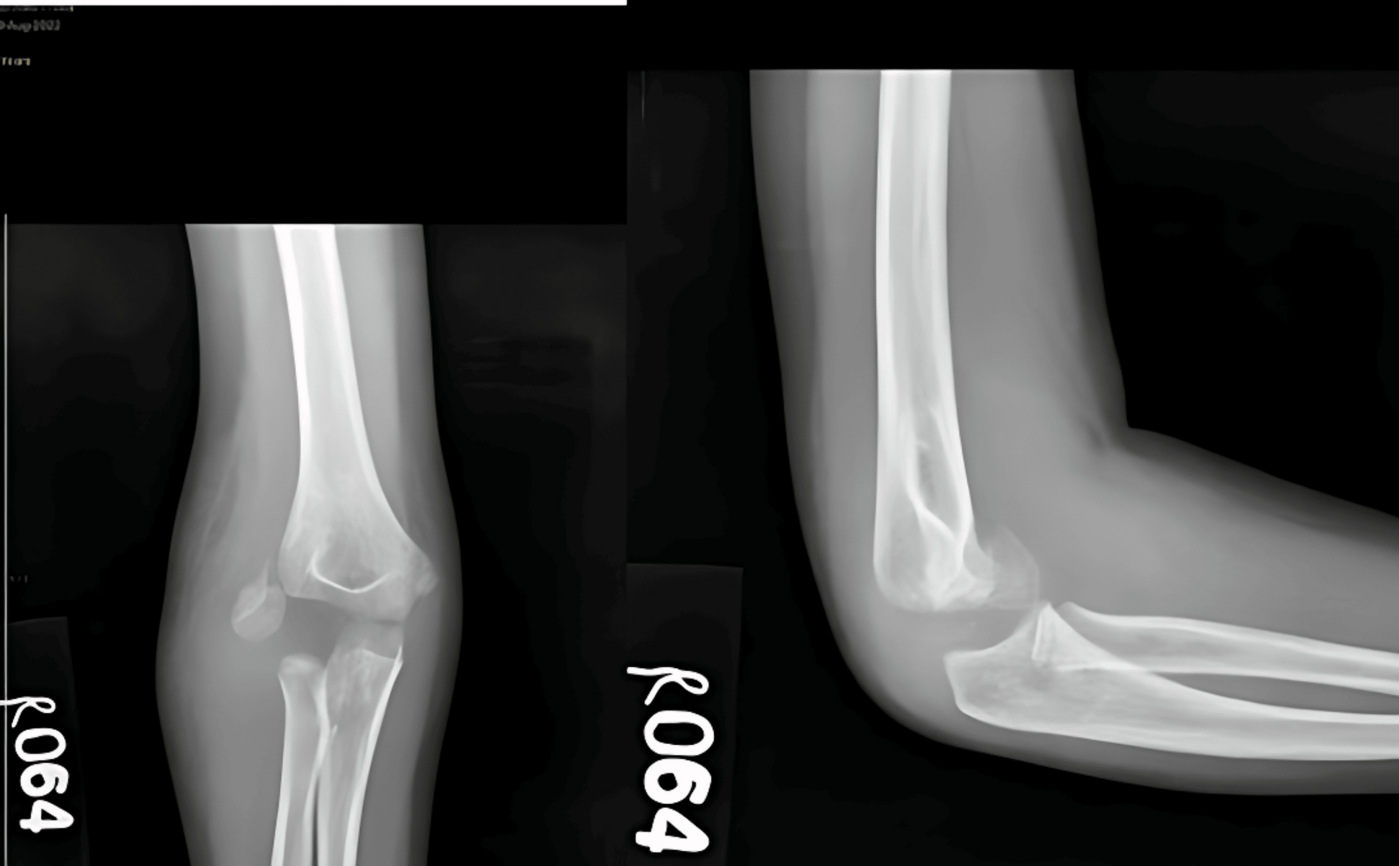
Preoperative X-ray of case 2 showing lateral condyle humerus fracture

**Figure 5:**
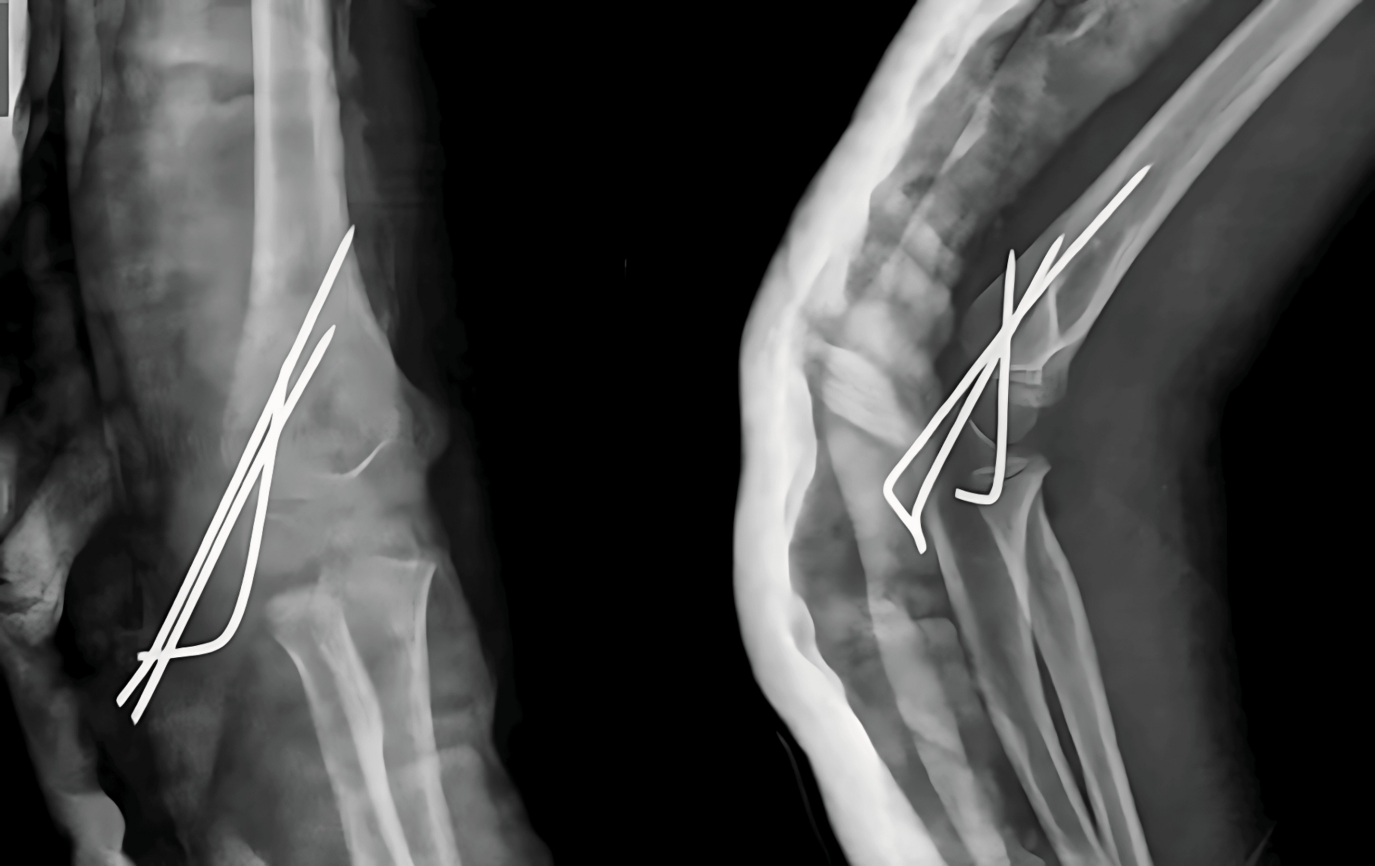
Postoperative X-ray of case 2

**Figure 6:**
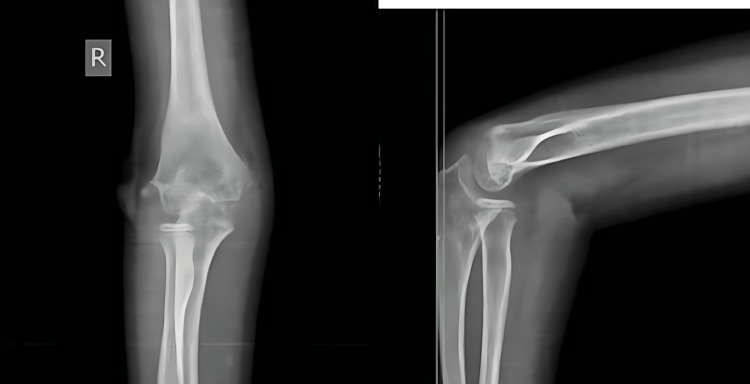
Postoperative X-ray of case 2 after implant removal

A five-year-old girl presented with a painful and deformed left elbow, which had persisted for 1.5 months following a fall during play. Surprisingly, the patient had not sought any medical treatment after the fall and had instead applied a local pain-relieving lotion recommended by a traditional bone setter. Unfortunately, the application of this lotion resulted in soft tissue irritation and maceration, ultimately leading to exposed bone from the lateral aspect of the deformity. Despite these complications, there were no signs of neurovascular deficit in the affected extremity. Radiological assessments, including anteroposterior and lateral radiographs and a CT scan, revealed a Jakob type III lateral condyle humerus fracture (Figure 4). Recognizing the need for intervention, open K-wiring of the lateral condylar fracture was performed three days after the patient's admission. The procedure followed a standard lateral approach to the distal humerus and elbow joint, allowing for direct visualization of the fracture, which was meticulously anatomically reduced and stabilized. To maintain the reduction, three smooth Kirschner wires (K-wires) were inserted in a lateral-to-medial and distal-to-proximal direction (Figure 5). The positions of these K-wires were diligently confirmed through fluoroscopic examination in both anteroposterior and lateral planes, and they were subsequently trimmed and left beneath the skin. Following the operation, the patient's elbow was immobilized in an above-elbow back slab. A comprehensive clinico-radiological follow-up was initiated at one and two weeks postoperatively, consistently demonstrating no loss of reduction. At six weeks post-operation, the back slab was removed, and elbow physiotherapy was initiated. The K-wires were safely removed under general anesthesia at nine weeks postoperatively (Figure 6). After two months of dedicated physiotherapy, the patient had regained normal elbow function with no observable alterations in the carrying angle. The patient's recovery was assessed at six months using the Broberg and Morrey score, which yielded a good result of 85 points. This case highlights the successful management of a delayed presentation of a compound grade 3 lateral condyle humerus fracture in a pediatric patient, resulting in a positive clinical outcome and functional recovery.

Case 3

**Figure 7:**
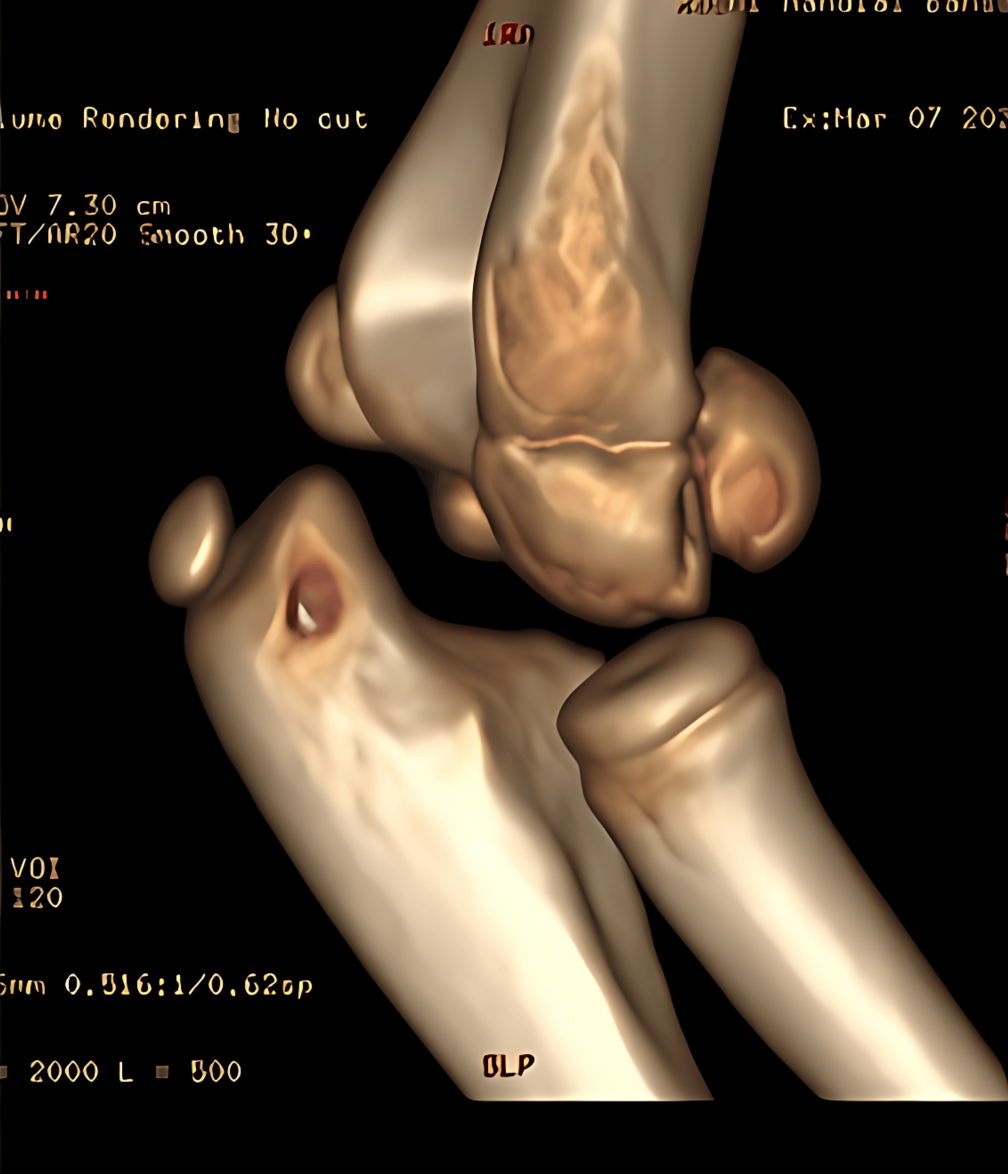
Preoperative CT scan of case 3 showing capitellum fracture

**Figure 8:**
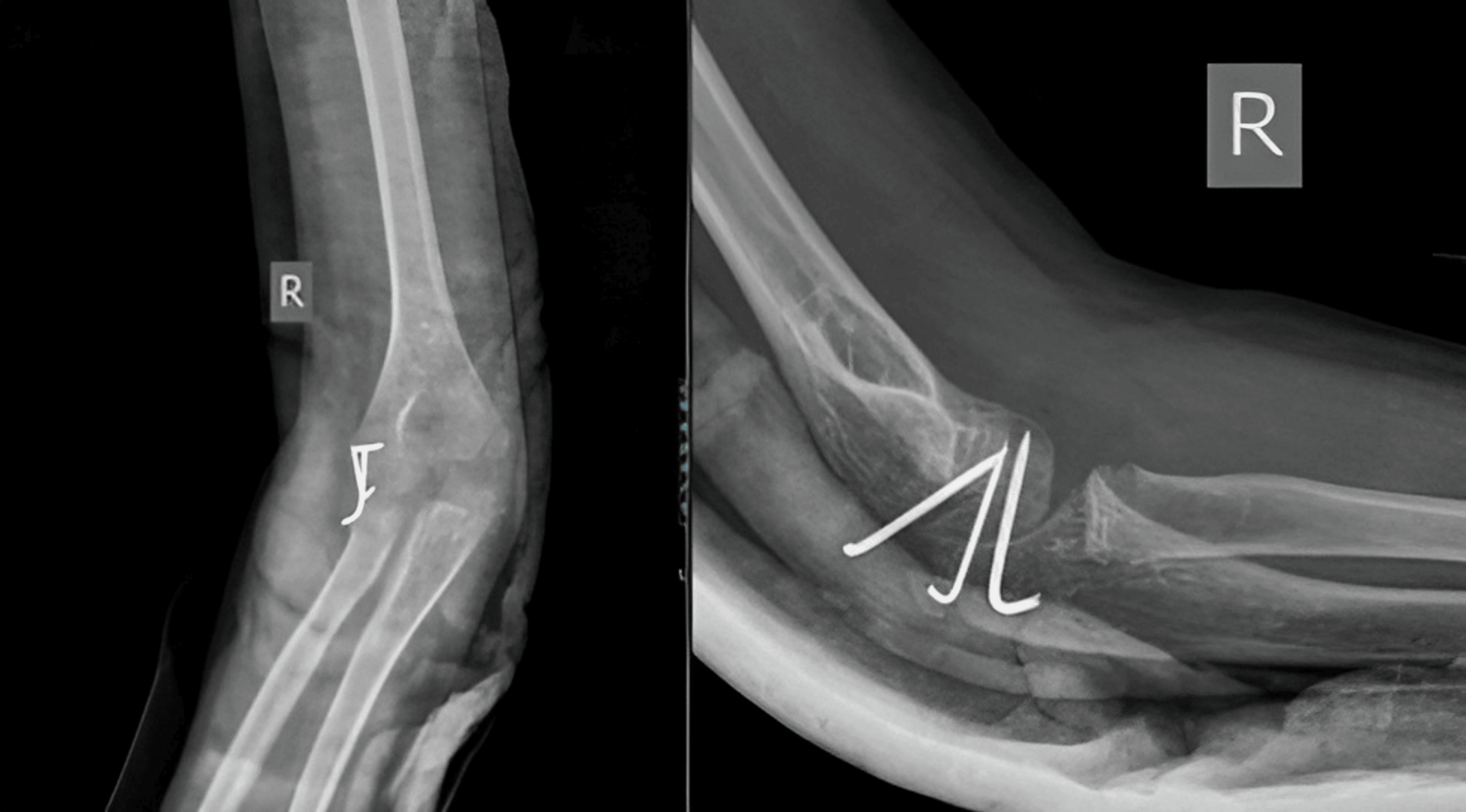
Postoperative X-ray of case 3

An 11-year-old girl had a history of a fall on her partially flexed right arm while going to school. She complained of pain and swelling in her right elbow with limitation of range of movement. She took some local treatment, and her elbow was splinted in 90° flexion. On examination, the elbow was found to be tender, swollen, and bruised. She would not allow either pressure or active movement of the joint. CT scan of the elbow revealed an anteriorly displaced type I (Hannis-Steinthal) capitellar fracture of the elbow joint (Figure 7). Open reduction and internal fixation (ORIF) was done with Kirschner wire through a lateral approach, taking care not to injure the posterior interosseous nerve. The fracture was reduced anatomically under vision and held with a pointed tenaculum, and K-wires were passed to hold the fracture fragment (Figure 8) in such a way that the trajectory of the K-wire lies outside the skin incision. Postoperatively, the elbow was held in 90° flexion with neutral position of the forearm, and she was discharged on the third postoperative day. She was reviewed four weeks postoperatively, at which time the splint was removed. Check X-rays showed no alteration from the immediate postoperative radiographs. Gentle elbow range of motion was started after splint removal. At six weeks postoperatively, a further check X-ray revealed the operative position to have been maintained, and elbow therapy was stepped up. She had an elbow joint range of motion sufficient to allow reasonable activities of daily living. The Kirschner wires were removed eight weeks postoperatively after fracture consolidation. By the time of her last follow-up at 18 weeks, she had 30°-130° flexion and extension with 70° pronation-supination. There was no evidence of avascular necrosis (AVN) of the fractured fragment, and the elbow was pain-free.

Case 4

**Figure 9:**
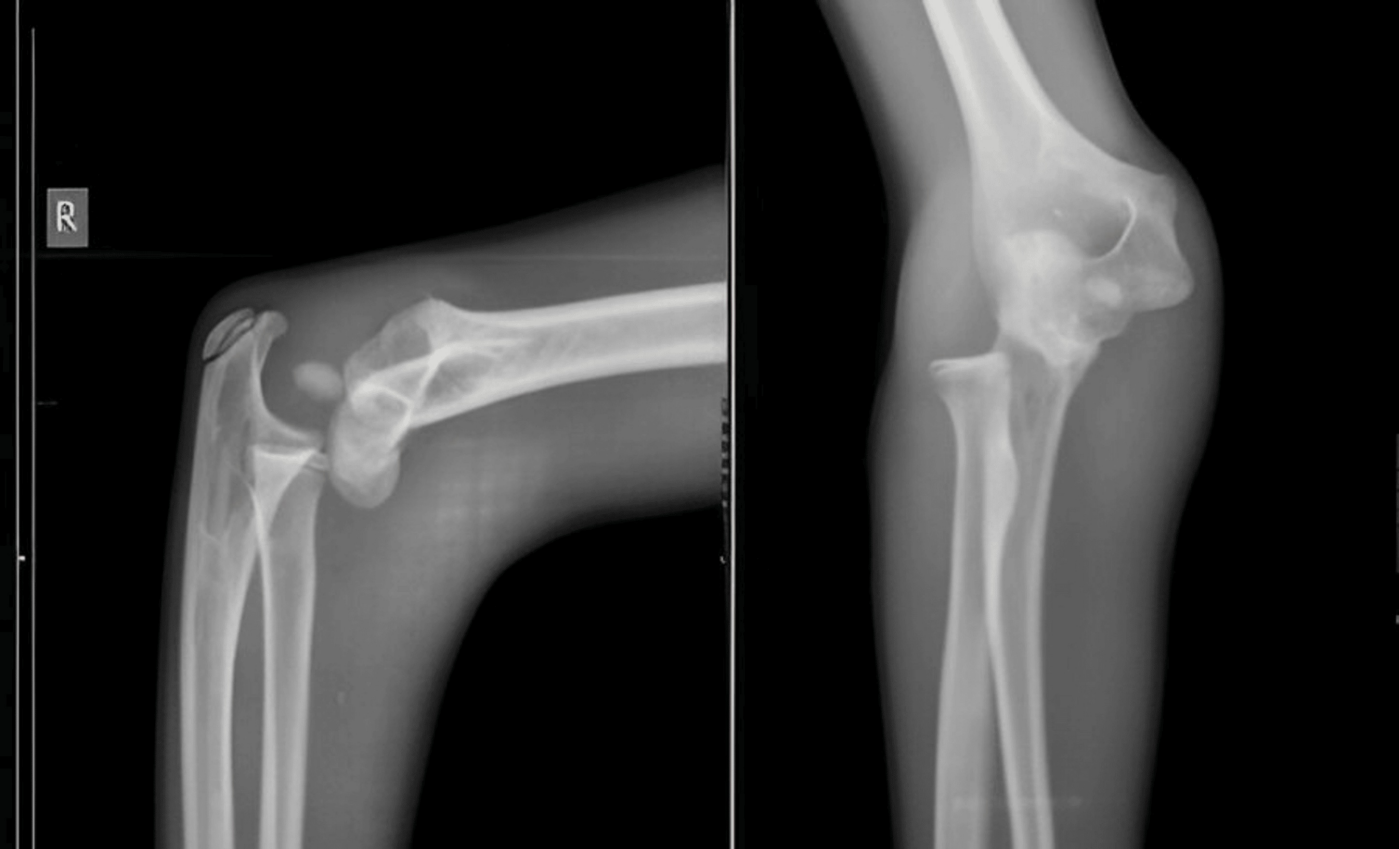
Preoperative X-ray of case 4 showing fracture dislocation of the right elbow

**Figure 10:**
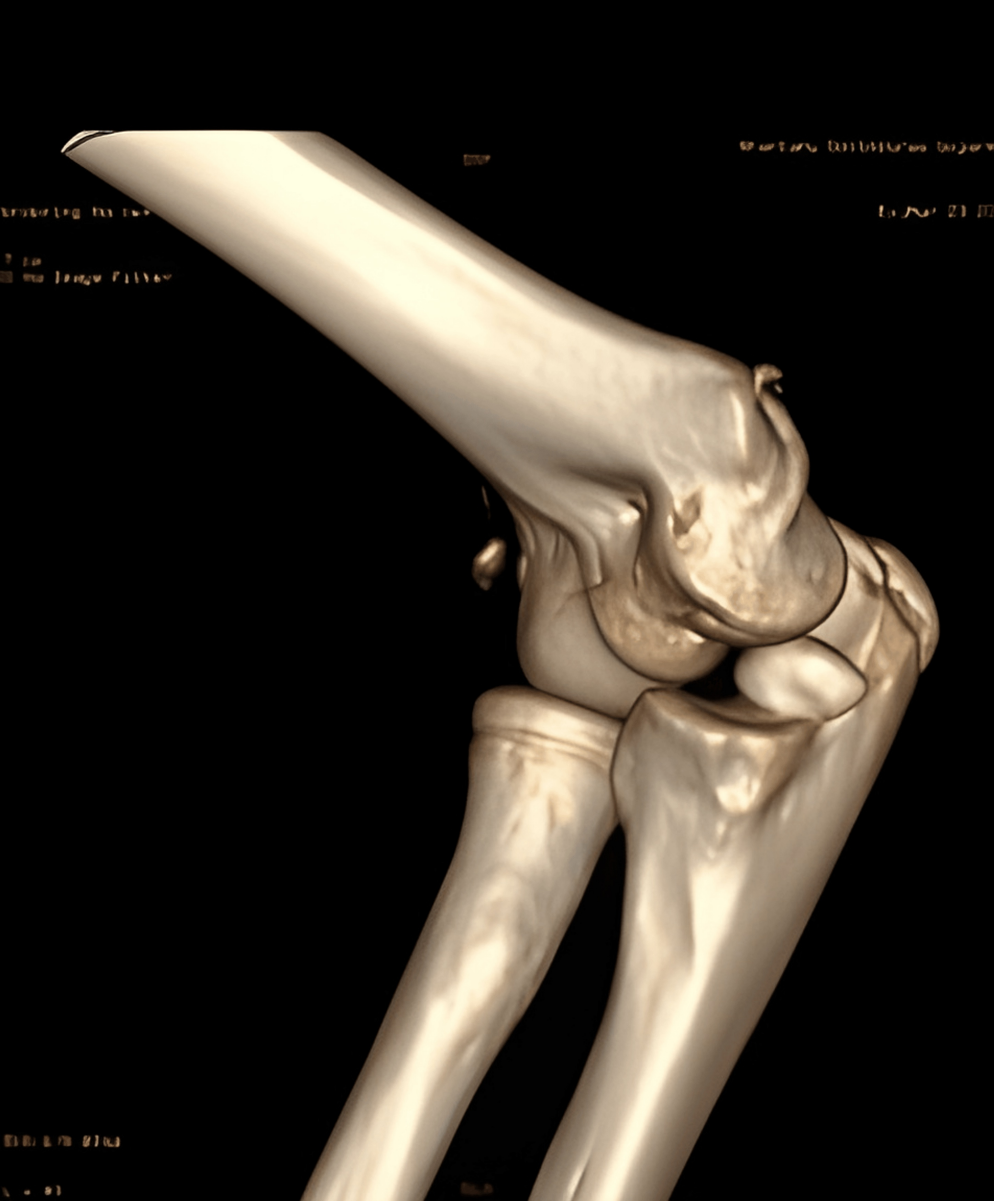
Preoperative CT scan of case 4 showing incarcerated fragment of the medial epicondyle

**Figure 11:**
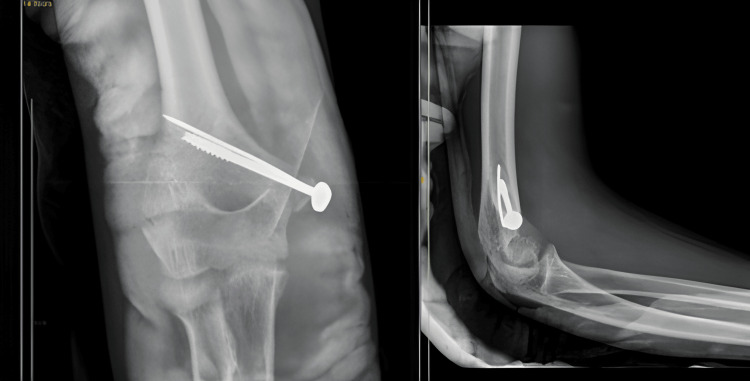
Postoperative X-ray of case 4

A 14-year-old boy arrived at our medical facility after experiencing a fall onto his right dominant hand while playing. He reported immediate pain, swelling, and limited movement in his right elbow. Upon examination, we observed joint deformity and swelling, with tenderness along the inner joint line upon palpation. Both voluntary and assisted movements of the elbow were restricted. An X-ray of the right elbow revealed posterior elbow dislocation with a fractured bone fragment from the inner part of the humerus trapped within the joint (Figure 9). A CT scan was done (Figure 10). Although we successfully reduced the elbow dislocation under mild sedation, the bone fragment remained lodged within the joint. Four days later, the patient underwent surgery to open up the elbow joint and fix the fractured bone fragment using screw and K-wire (Figure 11). During the procedure, we protected the ulnar nerve and covered the nerve with surrounding tissue to prevent irritation from the screws. Following surgery, the elbow was immobilized in a splint with a 90° flexion. The wound healed without complications, and physical therapy began three weeks later to restore elbow movement.

## Discussion

In our case series of rare elbow injuries, we reported a case of a Monteggia equivalent fracture, which was a segmental ulna fracture with a displaced fracture of the radial head along with a distal radius fracture. Certain similar rare injuries have been reported by Su et al. in their article "The diagnosis and treatment of a special rare type of Monteggia equivalent fractures in children" [6]. The treatment approach is akin to that used for Monteggia fractures. Initially, the focus was on restoring and stabilizing the length of the ulna and then addressing the fracture of the radial neck. Most fresh Monteggia fractures in children can achieve good outcomes through closed reduction and immobilization with plaster. Conversely, the majority of Monteggia equivalent fractures in children necessitate surgical intervention to avoid a poor prognosis. Ensuring that the proximal radius is anatomically realigned is crucial for its proper function [6]. The management of radial neck fractures continues to spark debate, with many authors favoring minimally invasive approaches. However, opting for open reduction carries the risk of complications including premature closure of the proximal radius epiphysis, elongation of the radial head, ischemic necrosis of the radial head epiphysis, and impaired elbow function [7].

Children rarely experience traumatic elbow dislocation, accounting for only 3%-6 % of all elbow injuries. Intra-articular entrapment of the fracture fragment in the elbow joint is observed in 5%-18% of elbow dislocation cases [8].

We reported one case of elbow dislocation where the fractured medial epicondylar piece was entrapped within the elbow joint, making the elbow joint unstable. Such injuries in children are rare, and few similar cases of incarcerated medial epicondyle within the elbow joint have been reported in the literature. Identifying an incarcerated epicondyle fracture fragment on a radiograph can be challenging due to potential overlap with the distal humerus metaphysis or confusion with ossification centers. As a result, additional imaging may be necessary. Failure to identify this can lead to limited elbow mobility and increased risk of ulnar nerve injury [9]. The operative intervention aims to achieve stable fixation, facilitating prompt elbow mobilization and averting deformity or stiffness. Various surgical interventions include fragment excision and sutures, closed or open reduction with Kirschner wire fixation, open reduction and suture fixation, and open reduction and screw fixation [10]. There are reports of non-union with only K-wire fixation. Therefore, in our case, the fracture was fixed with two K-wires temporarily, and one of the K-wires was replaced with cancellous cannulated screw after confirming acceptable reduction under C arm. Capitulum fractures, although uncommon, account for less than 1% of elbow fractures and have been documented to occasionally elude detection on plain radiographs [11]. During adolescence, as the capitellum undergoes growth and ossification, it becomes more prone to coronal shear injuries. Capitellar chondral shear injury, a less common variant of coronal shear fractures of the capitellum, involves a solitary fracture of the articular cartilage rim [12,13]. Joint stiffness and instability pose immediate risks following capitulum fractures, while the long-term concern includes the development of post-traumatic osteoarthritis [14]. Different methods of fracture fixation are accessible, including headless screw fixation, bioabsorbable pins, and suture fixation [15]. In our case, we performed an open reduction of the fracture using a lateral approach, meticulously realigning the articular fragment. We then compressed it using a pointed towel clip and secured it with three K-wires inserted from the posterior to the anterior direction. The K-wires provided stable fixation in multiple planes, and their removal after union was straightforward. We opted not to use permanent hardware such as Herbert screws as the removal of implant is more difficult.

Neglected fractures of the lateral humeral condyle (LHC) are fractures that are either unrecognized or misdiagnosed and present more than three weeks after the injury. Misdiagnoses can occur in as many as 17% of cases. The management of these neglected LHC fractures remains a topic of debate [16]. Numerous treatment options have been suggested, such as nonoperative approaches [17], open reduction and internal fixation (ORIF) [18], "in situ" fixation (ISF) [18], anterior transposition of the ulnar nerve (ATUN), and corrective osteotomy (CO) [19]. In our case, we did open reduction and internal fixation with K-wire; the fibrous tissue was debrided, and compression at the fracture site was achieved using a towel clip. Currently, there is a lack of sufficient reporting on treatment outcomes for neglected LHC fractures in children. Delayed surgery poses an elevated risk, potentially compromising the blood supply to the fragment, leading to avascular necrosis (AVN) and reduced elbow mobility [16].

## Conclusions

To conclude, managing elbow fractures in the young requires careful navigation due to intricate anatomical features and the dynamic nature of bone development in this age group. The rarity of conditions such as capitulum and Monteggia equivalent fractures necessitates a diligent and methodical approach to treatment to avert persistent complications. Swift and accurate diagnosis, followed by accurate reduction and stable fixation, whether non-surgical or surgical, is essential for the best possible recovery and for reducing risks such as impaired blood supply to the bone or reduced range of motion. Increased awareness and enhanced documentation of outcomes for overlooked fractures of the lateral humeral condyle will prove invaluable in refining our approach and informing clinical choices in these complex cases.
